# Preparation and Chromatographic Application of β-Cyclodextrin Molecularly Imprinted Microspheres for Paeoniflorin

**DOI:** 10.3390/polym9060214

**Published:** 2017-06-09

**Authors:** Wei Zhang, Bofeng Wei, Shoujiang Li, Yueming Wang, Shaoyan Wang

**Affiliations:** 1 School of Chemical Engineering, University of Science and Technology, Anshan 114051, Liaoning, China; askdzw@ustl.edu.cn or askdzw@163.com; 2 Liaoning Provincial Key Laboratory of Fine Separation Technique, University of Science and Technology, Anshan 114051, Liaoning, China; askdwbf@163.com (B.W.); lnkdlsj@163.com (S.L.); lnkdwym@163.com (Y.W.)

**Keywords:** molecular imprinting, stationary phase, paeoniflorin, chromatographic application

## Abstract

The application of molecular imprinting technology in the separation and purification of active ingredients in natural products was widely reported, but remains a challenge. Enrichment and separation are especially limited. A surface imprinting technique was reported to synthesize molecularly imprinted microspheres (MIMs) in this article. With paeoniflorin (PF) as the template molecule, β-cyclodextrin (β-CD) and acrylamide (AA) as the functional monomers, and poly(glycidyl methacrylate, GMA) microspheres (P_GMA_) as the backing material. MIMs have been characterized by FTIR and FESEM. Adsorption experiments indicated the adsorption capacity of MIMs was superior to those comparative non-imprinted microspheres (NIMs) and the binding isotherm of MIMs was in good agreement with the two-site binding model. The baseline separation of PF and its structural analogue albiflorin (AF) were achieved on the new MIMs packed column. MIMs showed good affinity and efficiency for separation of PF and AF compared with those comparative NIMs. The approach of fabricating MIMs is simple, rapid, and inexpensive, and may shed new light on the application of MIMs as a liquid chromatography stationary phase to separate and analyze PF and AF from the Red peony root extracts.

## 1. Introduction

Paeoniflorin (PF, CAS: 23180-57-6) is a monoterpene glycoside, which mainly exists in radix paeoniae rubra and radix paeoniae alba [1]. It has been declared to have antioxidant [2], anti-free radical damage [3], anti-platelet aggregation [4], improve microcirculation [5], and immune regulation [6]. It is relatively non-toxic and has served in cosmetics and medicines. In recent years, the separation of PF from herbs has attracted much attention in medical research. The traditional adsorbent, silica gel [7,8], and macro-porous adsorption resin [9,10] have been employed to separate and purify PF. However, the separation procedure is tedious and inefficient, and the traditional adsorbent is limited because of poor affinity and selectivity. It is difficult to enrich and separate PF using the traditional method because of the low content and the complex matrix in Chinese herbs. It is necessary to develop a more efficient method to enrich and separate PF.

Molecular imprinting technique is a rapidly developing technique for preparing highly cross-linked polymeric materials that contain highly specific recognition sites and with predetermined selectivity to template molecules [11]. Thus, the resulting molecularly imprinted polymers (MIPs) have the characteristics of the intended target, selectivity, and the recognition [12]. Because MIPs can selectively rebind the template or its analogs from a matrix, they have been extensively applied in solid-phase extraction (SPE) [13] and chromatography [14]. Because most MIPs have been synthesized by bulk polymerization and the preparation process is wasteful and time-consuming. The obtained irregularly shaped particles based on screening with MIPs were applied in SPE and high performance liquid chromatography (HPLC). Thus, more molecularly imprinted methods to obtain uniformly sized molecularly imprinted microspheres (MIMs) have been developed, such as seed polymerization [15], suspending polymerization [16], precipitation polymerization [17], and emulsion polymerization [18]. It is difficult to obtain MIMs with high affinity binding sites and selectivity with the preparation methods mentioned above, even those methods have significantly improved the adsorption properties of MIMs.

β-Cyclodextrin (β-CD) was a kind of cyclic oligosaccharide with a hollow cylindrical structure it has a hydrophilic exterior and an internal hydrophobic cavity [19]. The various kinds of inter-molecular interactions (van der Waals’ force, hydrophobic interaction, electrostatic affinity, dipole–dipole interaction, and hydrogen bond interaction) exist in β-CD and its derivatives, and of those interactions to entrap hydrophobic molecules. In recent years, β-CD has been widely used in the preparation of MIPs [20,21]. Despite this, most β-CD MIPs are prepared by bulk polymerization; however, spherical β-CD MIPs have been reported [22].

In this paper, MIMs for PF were prepared by surface imprinting [19]. Uniformly sized poly(glycidyl methacrylate, GMA) microspheres (P_GMA_) were invoked as the backing material, β-CD and AA as the functional monomers, and PF as the template molecule. The performance of MIMs was evaluated by adsorption experiments and chromatographic experiments. The MIMs exhibited excellent PF chromatographic performance, such as good separation of PF and its structural analogue albiflorin (AF) and larger adsorption capacity. This research might provide a new approach to the further separation and purification of natural products from natural product extracts.

## 2. Experimental

### 2.1. Instruments and Reagents

Characterization of the MIMs were carried out using Fourier transformed infrared (FTIR) spectroscopy (Nicolet IS10, Thermo Fisher, Waltham, MA, USA). The morphologies and structures of the MIMs were characterized by field emission scanning electron microscopy (FESEM, operated at 5.0 kV, Oberkochen, Germany). LC-10AT system (Shimadzu, Kyoto, Japan) was used for chromatographic analyses. Chromatographic packing system (HY-HPLC-1, Beijing, China) was used for packing column with MIMs and NIMs. A V-sorb 2800P surface area and pore size analyzer (Gold APP Co., Beijing, China) was used to measure physical adsorption parameters.

**Figure 1:**
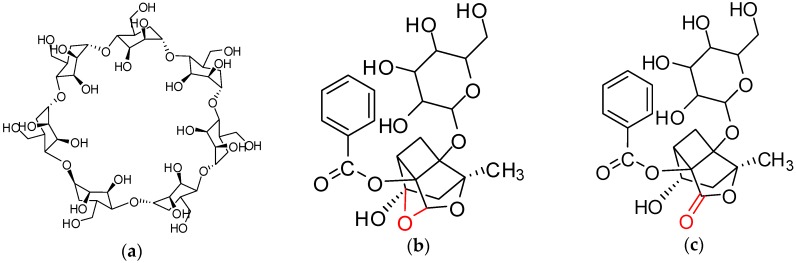
Structure of β-cyclodextrin (β-CD), paeoniflorin (PF), and albiflorin (AF): (**a**) β-CD; (**b**) PF; (**c**) AF.

PF and albiflorin (AF) (purity > 98%) were obtained from Shenzhen ziker Biological Technology Co. Ltd. (Shenzhen, China). β-CD and sodium hydrogen (NaH) were obtained from Tianjin Kermel Chemical Co. Ltd. (Tianjin, China). β-CD was recrystallized with distilled water before using. Structures of β-CD, PF, and AF are shown in Figure 1. Acrylamide (AA), styrene, PVP (K-30), and acetonitrile were purchased from Sinopharm Chemical Reagent Co. Ltd. (Shanghai, China). Ethyl glycol dimethacrylate (EGDMA), glycidyl methacrylate (GMA), poly(vinyl alcohol)-1788 (PVA-1788), and hydroxy ethyl methacrylate (HEMA) were obtained from Guangzhou Zhongye Chemical Co. Ltd. (Guangzhou, China). EGDMA was washed with 10% sodium hydroxide solution, saturated sodium chloride solution, and washed with distilled water and then dried with anhydrous sodium sulfate before using. 2,2′-Azobisisobutyronitrile (AIBN) was purchased from Shanghai SHV Chemical Co. Ltd. (Shanghai, China) and was purified by recrystallization with ethanol before polymerization. The other used chemical reagent was of analytical grade.

### 2.2. Synthesis of MIMs

**Figure 2:**
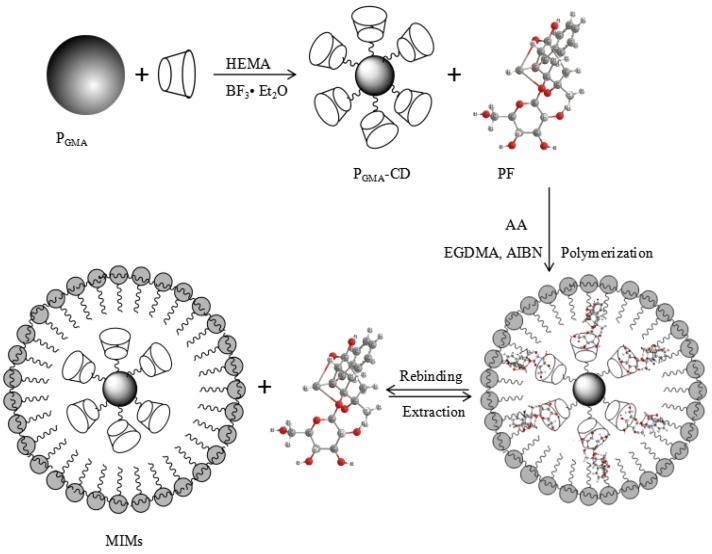
Scheme for preparation of poly(glycidyl methacrylate) microspheres (P_GMA_)-cyclodextrin (CD) and molecularly imprinted microspheres (MIMs).

MIMS were prepared with β-CD and AA, and P_GMA_ were used as the support matrix. The scheme for preparation of MIMs is shown in Figure 2.

#### 2.2.1. Synthesis of P_GMA_-CD

Uniformly sized P_GMA_ was prepared by the suspension polymerization method according to the reference [23].

The first step, P_GMA_-CD, was synthesized with β-CD and HEMA. In the process of preparation for P_GMA_-CD, 6.72 g (6.0 mmol) of β-CD was dissolved in 120 mL of anhydrous dimethylformamide (DMF) to which 0.50 g of NaH was added. The mixture was stirred at 30 °C in a water bath until no gas was emitted, and was filtrated to remove excessive NaH. Seven grams of P_GMA_ was added into the mixture, and the mixture was mechanically stirred at 30 °C in a water bath for 4 h. The solution temperature was increased by 10 °C every 30 min until 80 °C; then, the solution was mechanically stirred at 80 °C for 8 h under nitrogen protection.

In the second step, after the first reaction, the solution was naturally cooled to 40 °C, and a mixture solution containing 2.0 g of HEMA, 20 mL of DMF, and 40 μL of BF_3_·Et_2_O was added swiftly. The mixture was stirred at 40 °C for 12 h. The P_GMA_-CD was obtained of β-CD and the vinyl group was bonded on the surface of microspheres. P_GMA_-CD microspheres were washed with DMF, methanol, distilled water, and acetone, and dried at 30 °C, respectively. The determination method of immobilized β-CD [24]: The immobilized β-CD support (25.0 mg) was hydrolyzed in 15 mL of 0.5 mol/L H_2_SO_4_ at 100 °C for 8 h; then, the hydrolysate was made up to 50 mL with water, and the glucose content of hydrolysate was determined according to the method described in reference [25]. The amount of β-CD bonded on PGMA can be calculated by the following formula:

(1) $$A_{\left(\beta -\mathrm{CD}\right)}=\frac{C\times 50\times 1000}{180\times 7\times W}=39.7C/W$$

where *A* (μmol/g) is the amount of β-CD bonded on P_GMA_, *C* (g/mL) is the glucose concentration in hydrolysate, and *W* (mg) is the amount of immobilized β-CD support.

#### 2.2.2. Synthesis of MIMs

The mixture containing 50.0 mg (104.9 μmol) of PF, 65.0 mg (914.5 μmol) of AA, 245 mg (1.25 mmol) of EGDMA, 20 mg of AIBN, and 50 mL of dimethylsulfoxide was added to a 100 mL three-neck flask that contained 3.0 g of P_GMA_-CD microspheres. The mixture was stirred gently (60 rpm) under nitrogen protection for 8 h. Then, the flask was stirred (600 rpm) in a water bath at 65 °C for 8 h under constant nitrogen protection. The obtained MIMs were washed with distilled water and methanol to remove the template molecule and residual reagent.

Non-molecularly imprinted microspheres (NIMs) were prepared in the same way without the addition of PF for comparison.

Three kinds of MIMs and their comparative NIMs were prepared under different compositions, which are shown in Table 1.

### 2.3. Adsorption Experiments

Adsorption of PF from an aqueous solution (30% methanol) was investigated in batch experiments. The amount of MIMs, the adsorption isotherm, and the kinetics of the fabricated MIMs were determined. The adsorption capacity was calculated using the following equation:

(2) $$Q=\frac{\left(C_{0}-C_{e}\right)\times V}{M}$$

where *Q* (mg/g) is the amount of total adsorption of PF, *C*_0_ and *C*_e_ are the initial and equilibrium concentration of PF in solution (mg·L^−1^), respectively, *V* (L) is the volume of the solution, and *M* (g) is the weight of the polymers. The imprinting factor (IF) was calculated according to IF = *Q*_MIMs_/*Q*_NIMs_.

Fifty mg of MIMs and NIMs were placed in a tube and conditioned with 10 mL of 10 μmol·mL^−1^ PF solutions (30% methanol). The tube was incubated in a shaken bed at 25 °C. The solutions were analyzed by HPLC at different time intervals, and each analysis was repeated three times.

Scathard analysis was used to estimate binding characteristics of MIMs-1 and NIMs-1 according to the Scathard equation:

(3) QCe=(Qmax−Q)KD

where *K*_D_ is the equilibrium dissociation constant; *Q*_max_ is the apparent maximum number of binding sites; *C*_e_ is the equilibrium concentration of PF.

Fifty mg of MIMs or NIMs were placed in a tube and conditioned with 10 mL of 1.0–10 μmol·mL^−1^ solutions of PF (each solution concentration increased by 1.0 μmol·mL^−1^). The tube was incubated in a shaken bed at 25 °C for 80 min. The solutions were analyzed by HPLC, and each analysis was repeated three times.

For the polymers containing two types of binding sites, Scatchard analysis does not consider the contribution of the higher affinity binding sites and the lower affinity binding sites for the binding capacity of imprinted polymers at high concentrations and low concentrations of PF, respectively. Thus, the binding parameter values obtained by Scatchard analysis are inaccurate in the adsorption polymers that have two types of binding sites. The other equilibrium binding equation can be used to describe the adsorption process of two sites according to the following equation.

(4) $$Q=\frac{Q_{\max1}C}{K_{d1}+C}+\frac{Q_{\max2}C}{K_{d2}+C}$$

where *Q* is the amount of PF being adsorbed onto MIMs-1 or NIMs-1, *C* is the free concentration of PF in solution, *Q*_max1_ and *Q*_max2_ are the apparent maximum adsorption capacity of the higher and lower affinity binding sites, respectively, and *K*_d1_ and *K*_d2_ are the corresponding equilibrium dissociation constants of MIMs-1. *Q’*_max1_ and *Q’*_max2_ are the apparent maximum adsorption capacity of the higher and lower affinity binding sites, respectively, and *K’*_d1_ and *K’*_d2_ are the corresponding equilibrium dissociation constants of NIMs-1.

Fifty mg of MIMs or NIMs were placed in a tube and conditioned with 10 mL of 1.0–10.0 μmol·mL^−1^ PF solutions (each solution concentration increased by 1.0 μmol·mL^−1^). The tube was incubated in a shaken bed at 25 °C for 80 min. The solutions were analyzed by HPLC, and each analysis was repeated three times.

The selectivity of the fabricated MIMs and NIMs towards PF was studied by studying the adsorption of the MIMs and NIMs towards AF, the analogue of PF. 50 mg of MIMs and NIMs were placed in a tube and conditioned with 10 mL of 10.0 μmol·mL^−1^ PF solutions and 10 mL of 10.0 μmol·mL^−1^ AF solutions. The tube was incubated in a shaken bed at 25 °C for 80 min. The solutions were analyzed by HPLC, and each analysis was repeated three times.

The analyses of PF were performed using the LC-10AT system. Chromatographic separations were performed on a Zorbax-ODS column (150 mm × 4.6 mm, 5 μm). The UV detection wavelength was 230 nm. The mobile phase was a mixture of methanol–water–acetic acid (30:70:0.06, *v*/*v*/*v*) and the flow rate was 1.0 mL·min^−1^. A calibration curve was obtained to facilitate quantitative determination of PF, which is *y* = 207.8808 *x* + 4.9801 (2.0–20.0 μmol·mL^−1^, *R*^2^ = 0.9994).

### 2.4. Chromatographic Experiments

MIMs or NIMs were packed in a stainless steel column (150 mm × 4.6 mm) by a dilute slurry column-packing method with HY-HPLC. The solvent of the packing column was isopropyl alcohol. The packed column was washed with the mobile phase at the flow rate of 0.3 mL·min^−1^ until the stable baseline was obtained. Analyses of MIMs and NIMs were performed with an LC-10AT system consisting of an LC-10ATvp and SPD-10A UV detector. Methanol of uracil was used to estimate the dead time (*t*_0_). *t*_R_ was the retention time, the retention factor (*k*) was calculated according to *k* = (*t*_R_ − *t*_0_)/*t*_0_, and the separation factor (*α*) was according to α = *k*_PF_/*k*_AF_.

## 3. Results and Discussion

### 3.1. Characterization of MIMs

**Figure 3:**
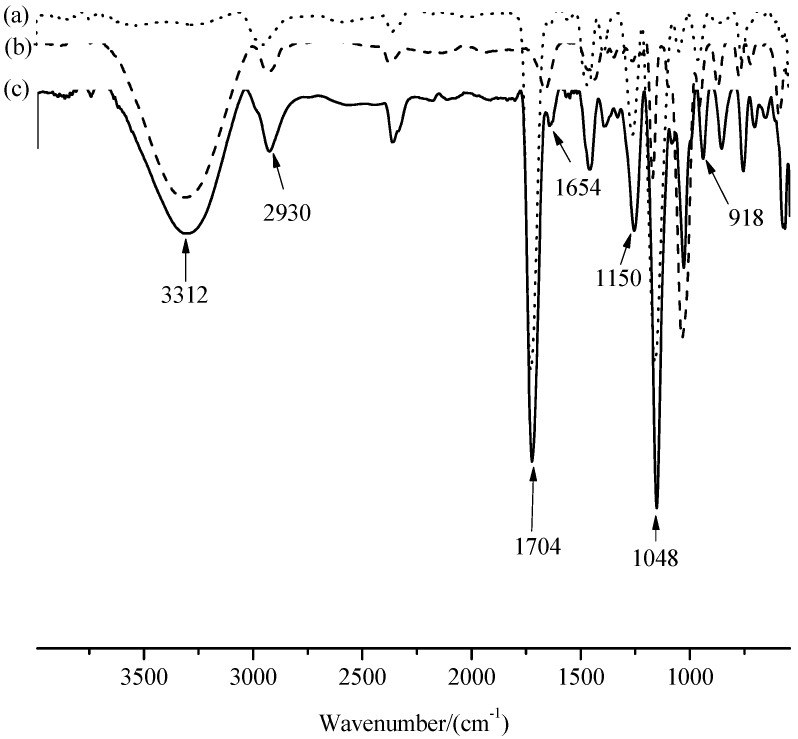
FTIR spectra of P_GMA_ (**a**), β-CD (**b**), and MIMs-1 (**c**).

Uniformly sized MIMs were prepared by surface imprinting. The IR spectra of PGMA (a), β-CD (b), and MIMs-1 (c) are given in Figure 3. The typical feature of epoxy group around 918 cm^−1^ disappeared, and the typical feature intensity of –CH_2_ (β-CD) and –OH (β-CD) increased around 2930 cm^−1^ and around 3312 cm^−1^, respectively, based on the analysis for the main characteristic peak’s position and intensity, which indicated that β-CD was bonded on P_GMA_. The stretching vibration peak of –C–O and –C–O–C around 1150 cm^−1^ and 1048 cm^−1^ were attributed to β-CD and β-CD polymers. The peak’s positions around 1704 cm^−1^ and 1654 cm^−1^ were attributed to the carbonyl groups of polyacrylamide, which indicates the successful preparation of MIMs-1. According to the analysis of IR spectrum, it was proved that the hydrophilic groups (hydroxyl groups and hydrogen bonds) and hydrophobic groups (methyl, methylene, etc.) both produce affinity adsorption with PF, which indicates that the hydrophobic interaction and the hydrogen-bonding interaction exist simultaneously in MIMs-1.

**Figure 4:**
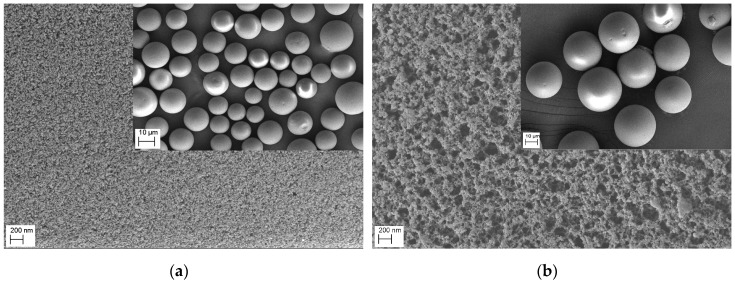
Scanning electron micrographs of P_GMA_ (**a**) and MIMs-1 (**b**).

FESEM pictures of P_GMA_ and MIMs-1 are shown in Figure 4. The diameters of P_GMA_ were about 25 μm, and it can be seen that the surface was monoporous, but the diameters of MIMs were about 30 μm and the surface was multi-porous. The change of the pore structure and the increase in diameter of the MIMs proved that β-CD and AA were bonded on the surface of P_GMA_. A V-sorb 2800P surface area and pore size analyzer was used to measure the specific surface areas, the total pore volume, and the average pore diameters of three kinds of MIMs and their comparative NIMs by the nitrogen adsorption method. As shown in Table 2, the total pore volume, the average pore diameters, and the specific surface areas of MIMs and NIMs increased compared with P_GMA_. The results showed that more complex β-CD polymers were grafted on the surface of the P_GMA_ microspheres. The specific surface area of MIMs was less than that of NIMs, and the average pore diameter of MIMs was greater than that of NIMs, as shown in Table 2. Thus, compared to NIMs, MIMs showed a more regular pore structure and a more uniform pore size. The difference in specific pore volume between MIMs and NIMs was small. Thus, the template molecule had an indistinct impact on the specific pore volume.

### 3.2. Adsorption Studies of MIMs

**Figure 5:**
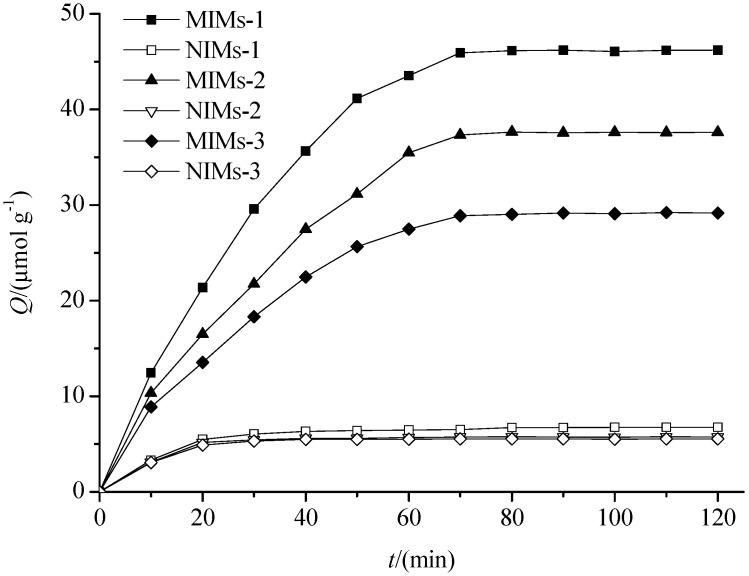
The adsorption dynamics curves of the MIMs and NIMs. Adsorption conditions: 10 mL of 10 μmol·mL^−1^ solutions (30% methanol) of PF with 50 mg of MIMs or NIMs.

The adsorption kinetic curves of PF on MIMs and NIMs are shown in Figure 5. The figure shows that the adsorption capacity of MIMs for PF was greater than that of the corresponding NIMs, and the adsorption capacity of MIMs-1 for PF was batter than that of MIMs-2 and MIMs-3. In addition, the adsorption equilibrium was achieved quickly. The main cause of the results was P_GMA_ was monoporous microspheres similar to silica microspheres, so the imprinted recognition sites were mainly bonded on the surface of MIMs. The IF of these three kinds of MIMs is shown in Table 3. The differences in IF revealed that MIMs-1 had a higher affinity when bonding with both β-CD and AA than the other two imprinted polymers microspheres when bonding with β-CD and AA separately; therefore, the hydrophobic interaction and the hydrogen-bonding interaction exist simultaneously in MIMs-1. This indicated that the structures of MIMs were different from NIMs because the structures of template molecules preserved in the MIMs and NIMs do not have the template structures, and the differences in the IF are mainly due to the “memory” recognition sites. Because β-CD has a hydrophilic exterior and an internal hydrophobic cavity, MIMs-1 has an inclusion effect on paeoniflorin, except the hydrophobic interaction and the hydrogen-bonding interaction. However, MIMs-3 and NIMs-3 have no inclusion effect on paeoniflorin, and the preparation methods of MIMs-2 (and NIMs-2) and MIMs-3 (and NIMs-3) were the traditional preparation methods of molecularly imprinted polymers with only one interaction. These results indicate a hydrophobic interaction combined with a hydrogen-bonding interaction, and the inclusion effect was stronger than both the hydrophobic interaction and the hydrogen-bonding interaction. Therefore, in the following studies, only MIMs-1 was investigated.

**Figure 6:**
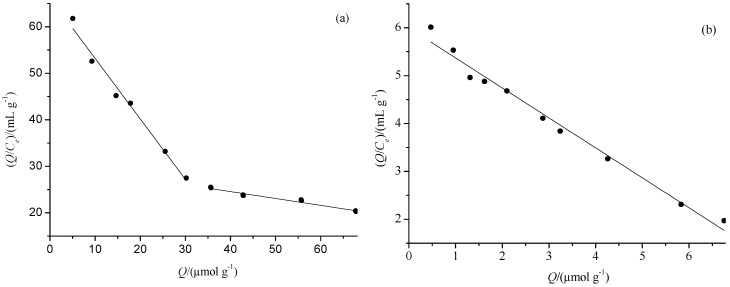
The Scatchard plots of MIMs-1 (**a**) and NIMs-1 (**b**) for PF.

The Scatchard dissociation curves of PF on MIMs-1 and NIMs-1 are shown in Figure 6. There were two discontinuous liners for MIMs-1, which indicated there were two types of affinity binding sites in MIMs-1. On the contrary, there was only one line for NIMs-1, which indicated there was one type of binding sites in NIMs-1. For MIMs-1, the regression equation was *Q*/*C*_e_ = −1.300 *Q* + 66.20 (r = 0.9816) for the higher affinity binding site, and the regression equation was *Q*/*C*_e_ = −0.148 *Q* + 30.61 (r = 0.9574) for the lower affinity binding site. Therefore, *K*_D_ and *Q*_max_ were 0.7692 mmol·g^−1^ and 50.92 μmol·g^−1^ for the higher affinity binding site, and 6.7568 mmol·g^−1^ and 206.83 μmol·g^−1^ for the lower affinity binding site, respectively, according to the two regression lines. For NIMs-1, the regression equation was *Q*/*C*_e_ = −0.626 *Q* + 5.9953 (r = 0.9831), and *K*_D_ and Q_max_ were 1.5974 mmol·g^−1^ and 9.577 μmol·g^−1^, respectively. The main reason for the difference of the Scatchard curves of MIMs-1 and NIMs-1 were the different recognition sites were preserved in the two kinds of polymers.

**Figure 7:**
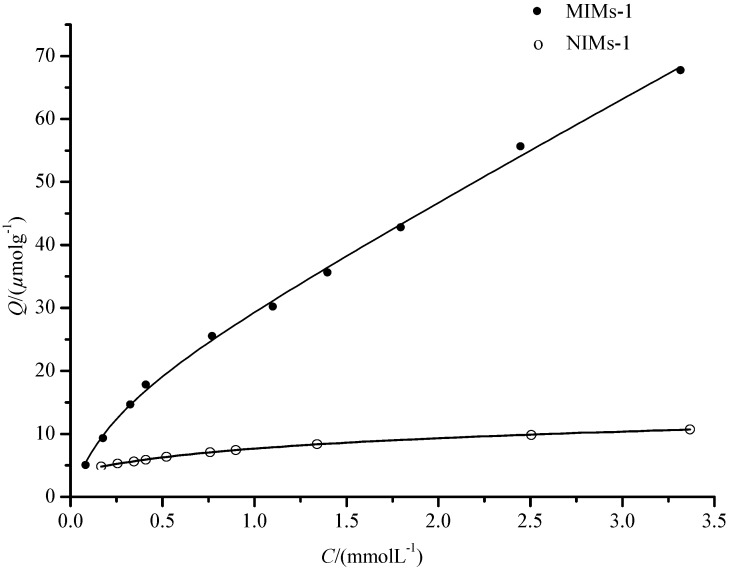
The fitting curve of equilibrium binding equation.

Meanwhile, the equilibrium binding fitting curve of PF on MIMs and NIMs was obtained by *Q* versus *C* according to Equation (4) and is shown in Figure 7. As shown in Figure 7, the fitting curve was in good agreement with the experimental points. For MIMs, *Q*_max1_, *Q*_max2_, *K*_d1_, and *K*_d2_ were 16.46 μmol·g^−1^, 595.85 μmol·g^−1^, 0.2453 mmol·L^−1^, and 36.98 mmol·L^−1^, and the correlation coefficient of the fitting curve was 0.9975. Moreover, for NIMs, *Q’*_max1_, *Q’*_max2_, *K’*_d1_, and *K’*_d2_ were 169.84 μmol·g^−1^, 7.21 μmol·g^−1^, 39.81 mmol·L^−1^, and 1.26 mmol·L^−1^, and the correlation coefficient of the fitting curve was 0.9997. The difference between *Q*_max_ and *K*_d_ indicated that the MIMs have a higher selective adsorption capacity (*Q*_max2_ > *Q’*_max2_) than NIMs, and NIMs have a higher non-selective adsorption capacity (*Q*_max1_ < *Q’*_max1_) than MIMs. This indicates that the values obtained by the equilibrium binding equation are more workable compared to the Scathard analysis.

**Figure 8:**
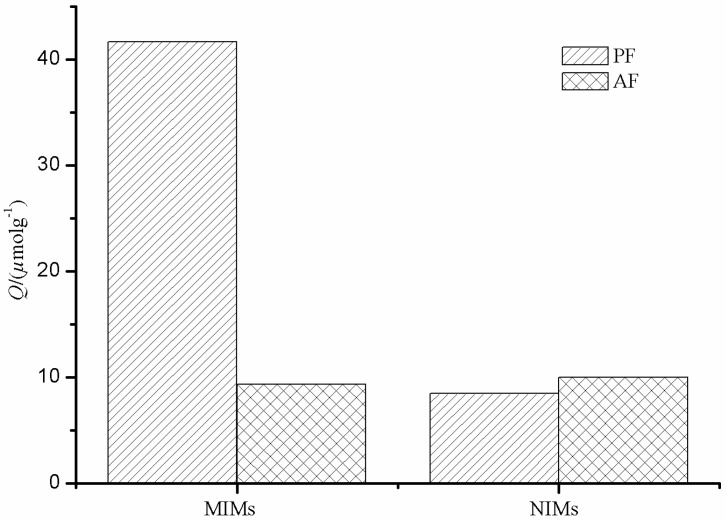
Selectivity study of PF and AF with MIMs and NIMs.

Selectivity of MIMs with regard to NIMs was studied, and the result is shown in Figure 8. As can be seen from Figure 8, MIM particles have a higher selective adsorption towards PF compared to AF, while the adsorption capacities of PF and AF by NIMs were almost similar to each other. Imprinting sites formed in the MIMs have the capability of distinguishing target molecules through their size, shape, and functional group distribution. However, there are non-imprinting sites in NIMs to adsorb PF and AF.

### 3.3. Chromatographic Analysis

**Figure 9:**
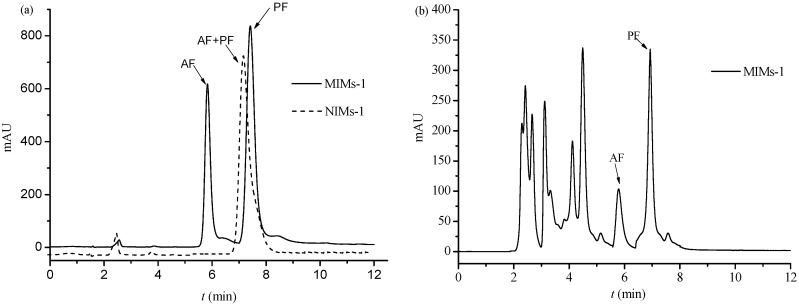
Chromatograms of PF and AF on MIMs-1/NIMs-1 (**a**) and Chromatogram of Red peony root extracts on MIMs-1 (**b**). LC conditions of MIMs-1/NIMs-1 (**a**,**b**): mobile phase: methanol–water–acetic acid (27:73:0.06, *v*/*v*/*v*); flow rate was 0.5 mL·min^−1^; extracting conditions of Red peony root extracts: 10 g of Red peony root powder was dissolved in 40 mL of 50% ethanol solution (v %), the extraction temperature was 50 °C, and the time was 3 h.

PF and its similar structure AF have analogous properties, and they are all monoterpene glycosides. Therefore, it is difficult to separate PF and AF using the traditional column chromatography. The feasibility of utilizing MIMs-1 as HPLC stationary phase for the separation of PF and AF was investigated. The chromatogram of PF and AF on the reversed phase columns with MIMs-1 and NIMs-1 as the stationary phase (as shown in Section 2.4), respectively, is shown in Figure 9a. As shown in Figure 9a, baseline separation was achieved on the imprinted column of PF and AF, and it could not be achieved on the non-imprinted column. The separation factor was 1.47 on the imprinted column. The main cause of the difference in separation on the two columns was the special recognition sites existing in the MIMs-1.

Generally speaking, the performance of the MIMs was strongly dependent on the solvent/eluent used in the adsorption/desorption processes, especially for non-specific interactions. The methanol/water mixtures were specifically selected in the testing of the materials after the experimental optimization (see [26]). The results indicate that the viscosity of ethanol was greater than that of methanol and acetonitrile at room temperature. Furthermore, the viscosity of ethanol was similar to that of methanol and acetonitrile at 50 °C. However, chromatographic separation is usually performed at room temperature. In addition, acetonitrile is expensive, and its elution capacity is similar to that of methanol. Therefore, methanol was chosen as solvent/eluent. The choice of methanol/water ratio was mainly the optimization result of chromatographic separation and retention time.

More complex components exist in the Red peony root extracts, except PF and AF. Therefore, it is more difficult to separate PF from the Red peony root extracts. The chromatogram of the Red peony root extracts on the imprinted column (as shown in Section 2.4) is shown in Figure 9b. It can be seen that there were many different constituents in the extracts. PF and AF could also be separated by baseline. These results indicate the possibility of applying MIMs-1 as the stationary phase to analyze or separate the Red peony root extracts.

## 4. Conclusions

To separate PF and AF, a new approach for the fabrication of the PF MIMs was presented by molecular imprinting. The modified MIMs exhibited a larger adsorption capacity (206.83 μmol·g^−1^), good recognition, and a good chromatographic separation performance. Compared with MIMs-2 and MIMs-3, which have single affinity binding sites, the MIMs-1, which has two types of affinity binding sites, has a higher affinity and selectivity for PF, and the adsorption isotherm is in good agreement with the two-site binding model. PF and AF can be separated at baseline on the imprinted column, with MIMs-1 as the stationary phase. The MIMs-1 could act as an adsorbent for separating Red peony root extracts. In addition, this fabrication procedure is simple, rapid, and inexpensive. We believe that this method may inform the separation of other natural product intermediates that have an analogue structure.

## Figures and Tables

**Table 1:** The preparative composition of different MIMs and non-imprinted microspheres (NIMs).

Polymer	PF (μmol)	β-CD ^a^ (μmol)	AA (μmol)	EGDMA (mmol)	AIBN (mg)
MIMs-1	104.9	102.9	914.5	1.25	20
NIMs-1	-	102.9	914.5	1.25	20
MIMs-2	104.9	102.9	-	1.25	20
NIMs-2	-	102.9	-	1.25	20
MIMs-3	104.9	-	914.5	1.25	20
NIMs-3	-	-	914.5	1.25	20

^a^ The content of bonded β-CD.

**Table 2:** Properties of P_GMA_, MIMs, and NIMs.

Polymer	Specific surface area ^a^ (m^2^·g^−1^)	Average pore diameter ^b^ (nm)	Specific pore volume ^c^ (mL·g^−1^)
P_GMA_	148.35	21.85	0.74
MIMs-1	240.38	35.58	0.85
NIMs-1	254.16	28.92	0.83
MIMs-2	234.84	33.47	0.84
NIMs-2	242.52	28.73	0.87
MIMs-3	227.81	31.83	0.83
NIMs-3	230.77	29.14	0.86

^a^ Measured by Brunauer–Emmett–Teller (BET) method; ^b^ Measured by Dubinin–Radushkevich (D-R) method; ^c^ Measured by the Barrett-Joyner-Halenda (BJH) method.

**Table 3:** Imprinting factor (IF) of three kinds of MIMs.

Polymer	Q ^a^	IF
MIMs-1	45.93	7.06
NIMs-1	6.51	
MIMs-2	37.33	6.53
NIMs-2	5.72	
MIMs-3	28.87	5.22
NIMs-3	5.53	

^a^ The amount of total adsorption of PF were measured at 70 min.
